# Prediction of post-operative complications among diabetic patients undergoing major surgery using biomarkers

**DOI:** 10.6026/973206300211543

**Published:** 2025-06-30

**Authors:** Jekee Patel, Parin Patel, Himarshi Upadhyay, Falak Saiyed, Huma Saiyad, Swarnim Rathod

**Affiliations:** 1 Department of Surgery, GMERS Medical College and Hospital, Vadnagar, Gujarat, India; 2 Department of General Surgery, Health 1 Super Specialty Hospital, Ahmedabad, Gujarat, India; 3 Department of Internal Medicine, GMERS Medical College and Hospital, Vadnagar, Gujarat, India; 4 Department of Medicine, NHL Municipal Medical College, Ahmedabad, Gujarat, India; 5 Department of Medicine, BJ Medical College, Ahmedabad, Gujarat, India; 6 House Officer cum Tutor, GMERS Medical College and Hospital, Vadnagar, Gujarat, India

**Keywords:** Diabetes mellitus, HbA1c, C-reactive protein, IL-6, Procalcitonin, Postoperative complications, Biomarkers, Surgical risk assessment

## Abstract

Evaluation of pre-operative HbA1c, CRP, IL-6, and procalcitonin levels among 150 diabetic patients undergoing major surgery is of interest. Elevated HbA1c (>8%) and CRP were strongly associated with postoperative complications such as wound infections and prolonged ICU stay. IL-6 and procalcitonin show moderates predictive accuracy for systemic inflammation. Biomarker profiling proved useful for identifying high-risk patients. Incorporating these tests can improve surgical planning and postoperative care.

## Background:

Diabetes mellitus (DM) is widely recognized as a significant risk factor for adverse perioperative outcomes, primarily due to its associated vascular and metabolic complications [1]. A growing body of literature suggests that diabetic patients undergoing surgical interventions are more susceptible to serious postoperative complications such as surgical site infections, delayed wound healing, cardiovascular events, and prolonged hospital stays [2, 3]. These adverse outcomes are largely attributed to the chronic hyperglycemic state seen in DM, which impairs immune responses, disrupts vascular integrity, and compromises tissue regeneration and repair mechanisms [4]. As surgical complexity increases, the need for accurate preoperative risk assessment tools becomes imperative, particularly in diabetic patients. Among the most commonly used biomarkers, glycated hemoglobin (HbA1c) has emerged as a strong predictor of postoperative prognosis, with studies indicating a significantly increased risk of complications when preoperative HbA1c levels exceed 8% [5]. In parallel, inflammatory markers such as C-reactive protein (CRP), interleukin-6 (IL-6), and procalcitonin (PCT) have shown potential in forecasting systemic inflammation and identifying patients at risk of poor surgical outcomes [6, 7]. Elevated levels of these biomarkers prior to surgery may reflect underlying inflammatory conditions, which are often undiagnosed but contribute to increased vulnerability to complications following surgery [8]. IL-6, in particular, is a pivotal cytokine that not only modulates the immune response but also plays a role in metabolic regulation. Its preoperative elevation has been linked to exaggerated postoperative inflammatory reactions, including sepsis and impaired wound healing [9]. Similarly, CRP-an acute-phase reactant synthesized by the liver-serves as a robust marker for identifying infections and adverse cardiac events in surgical patients [10]. The synergistic evaluation of IL-6 and CRP can help establish a patient-specific inflammatory profile, which is especially critical for diabetic individuals with compromised immune defenses [11]. Procalcitonin, a highly specific marker for bacterial infections is increasingly being investigated for its role in distinguishing between infectious and non-infectious postoperative inflammatory responses [12]. While its standalone predictive value remains under study, PCT demonstrates improved diagnostic utility when assessed in conjunction with IL-6 and CRP [13]. The use of a multimarker approach enhances perioperative risk stratification, enabling clinicians to tailor individualized therapeutic strategies. Diabetic patients with elevated HbA1c and concurrent increases in inflammatory biomarkers may particularly benefit from intensified glycemic control and prophylactic anti-inflammatory interventions before surgery. Timely biomarker profiling allows for early identification of potential complications, facilitating prompt clinical intervention to mitigate morbidity. As the global burden of diabetes continues to rise and the volume of surgical procedures increases, the development and validation of evidence-based risk prediction tools are becoming increasingly essential. Therefore, it is of interest to investigate the diagnostic power of HbA1c combined with CRP and IL-6 together with procalcitonin as predictors for postoperative complications within diabetic patients who need major elective surgical interventions.

## Materials and Methods:

The research studied adult type 2 diabetes mellitus patients across a tertiary care surgical facility from January 2023 to December 2024 in an observational prospective design. Three hundred adult patients diagnosed with type 2 diabetes mellitus underwent major elective surgeries under general anesthesia at multiple hospital centers. The research criteria selected adult patients between 30 to 75 years old who received a diabetes diagnosis during the last twelve months with American Society of Anesthesiologists physical statuses of II or III. Surgical emergency patients alongside people with active infections or malignancies were excluded from the study together with patients receiving immunosuppressive therapy and those affected by chronic inflammatory diseases. The evaluation process consisted of thorough investigation through patient history and physical examination as well as standard laboratory tests. Surgical patients received blood sampling for biomarker evaluation of glycated hemoglobin (HbA1c) and C-reactive protein (CRP) and interleukin-6 (IL-6) and procalcitonin prior to their 24-hour preoperative period. The assessment of IL-6 and procalcitonin depended on standardized enzyme-linked immunosorbent assay (ELISA) kits while CRP and HbA1c measurements happened through automated analysers. Surgical site infection (SSI) joined delayed wound healing together with sepsis and extended intensive care unit (ICU) stay as the monitored postoperative complications for 30 days. The Clavien-Dindo grading system served to categorize the encountered complications. The study used a structured case proforma for data recording. SPSS version 25.0 served as the platform for statistical data evaluation. Type I variables showed their values as mean ± standard deviation while type IV variables showed frequencies and percentages. We conducted the chi-square test together with the independent t-test for identifying associations between biomarker measurements and postoperative results. The research used logistic regression to discover independent factors that lead to postoperative complications. The study regarded p-values under 0.05 as indicating significant statistical results.

## Results:

A total of 150 diabetic patients undergoing major elective surgeries were included in the study. The mean age was 58.4 ± 9.7 years, with 60% males (n=90) and 40% females (n=60). The most common surgeries performed were abdominal (40%), orthopedic (30%), and cardiothoracic (30%). Preoperative mean HbA1c was 8.2 ± 1.1%, mean CRP was 12.6 ± 4.5 mg/L, IL-6 was 22.4 ± 7.8 pg/mL, and procalcitonin was 0.42 ± 0.15 ng/mL. Postoperative complications occurred in 52 patients (34.7%), with surgical site infection being the most frequent (20%), followed by delayed wound healing (8.7%) and prolonged ICU stay (6%). Patients who developed complications had significantly higher preoperative HbA1c (8.9 ± 0.9 vs. 7.8 ± 1.0, p<0.001), CRP (15.1 ± 3.8 vs. 11.2 ± 4.2, p=0.002), IL-6 (28.3 ± 6.7 vs. 18.9 ± 6.3, p<0.001), and procalcitonin (0.51 ± 0.11 vs. 0.37 ± 0.13, p=0.004) compared to those without complications (Table 1 - see PDF). Multivariate logistic regression identified elevated IL-6 (OR 2.14, 95% CI: 1.33-3.44), HbA1c >8% (OR 1.96, 95% CI: 1.12-3.45), and CRP >10 mg/L (OR 1.88, 95% CI: 1.05-3.12) as independent predictors of postoperative complications (Table 2 - see PDF). These findings suggest that elevated levels of preoperative inflammatory and glycemic markers are significantly associated with an increased risk of adverse postoperative outcomes in diabetic patients undergoing major surgery (Table 1 - see PDF and Table 2 - see PDF).

## Discussion:

The study proves that diabetic patients experience elevated postoperative complications from elevated preoperative readings of HbA1c, CRP and IL-6 and procalcitonin when undergoing major surgery. The research leads to the conclusion that biomarker screening enables medical teams to detect patients at high risk before surgery and develop appropriate postoperative management plans. The medical community accepts glycated hemoglobin (HbA1c) as a long-term glucose control indicator which demonstrates a proven link between elevated levels and inferior surgical outcomes that produce more infections with slow healing [1, 2]. Patient data demonstrated elevated postoperative complication rates among those with HbA1c superior to 8% showing parallel observations from existing literature that HbA1c controls under 8% promote positive surgical outcomes in orthopedic and cardiovascular operations and abdominal procedures [3, 4]. Systemic inflammation can be measured through the tested acute-phase reactant called C-reactive protein (CRP). The literature shows that CRP elevation before surgery helps identify surgical site infection risks and sepsis development especially during gastrointestinal and oncological procedures [5, 6]. Our research demonstrates that individuals presenting with CRP levels exceeding 10 mg/L face double the likelihood of adverse outcomes thus establishing its value as a mandatory screening tool before performing surgery on diabetic patients [7]. The pro-inflammatory cytokine interleukin-6 (IL-6) acts to control immune functions and aid tissue recovery processes. High IL-6 values indicate increased systemic inflammation thus leading to prolonged recovery times and greater medical problems [8]. Research has proven IL-6 works as an early warning indicator for postoperative inflammation since it detects fever among other problematic outcomes in critical and hospital surgical patients [9, 10]. Our research data confirms this hypothesis since postoperative complications became more likely when IL-6 measurements reached or exceeded 25 pg/mL. The biomarker procalcitonin serves as a distinct indicator that detects bacterial infection together with systemic inflammatory response in patients. The preliminary analysis found an association between PCT and complications but it failed to achieve statistical significance in the multivariate model which indicates limited independent capability for predicting postoperative complications in elective surgical patients [11, 12]. Previous research indicates that PCT performance improves when combining this test result with other biomarkers [13]. Widening risk stratification models with these biomarkers enables clinicians to detect vulnerable patients so they can decide on necessary interventions such as enhanced glycemic control as well as prehabilitation and modified surgical plans. Follow-up assessments of inflammatory markers enable physicians to observe complications postoperatively while helping them begin prompt intervention. The research has several promising outcomes although it carries specific limitations. The research drawbacks included a low number of study participants and data acquisition from only one healthcare facility therefore reducing the universal applicability of reported results. The tracking of complications after surgery extended to thirty days but did not account for results beyond this period. Future examination using broader and multihospital studies spanning extended observation periods needs to validate these results and establishes unified biomarker-based procedures for diabetic patients during surgery.

## Conclusion:

The levels of HbA1c as well as CRP and IL-6 biomarkers elevation immediately before surgery correlate strongly with higher post-operative complication risks in diabetic patients who undergo major operations. Pre-surgical evaluations using these biomarkers help discover high-risk patients prior to surgery to enable specific therapeutic measures that result in better perioperative results.
